# Bacteriocin Production Correlates with Epidemiological Prevalence of Phylotype I Sequevar 18 Ralstonia pseudosolanacearum in Madagascar

**DOI:** 10.1128/aem.01632-22

**Published:** 2023-01-05

**Authors:** Hasina Rasoamanana, Santatra Ravelomanantsoa, Marie-Véronique Nomenjanahary, Miharisoa-Mirana Gauche, Philippe Prior, Fabien Guérin, Isabelle Robène, Yann Pecrix, Stéphane Poussier

**Affiliations:** a University of Reunion Island, UMR PVBMT, Saint-Pierre, Reunion Island, France; b CENRADERU/FOFIFA, Antananarivo, Madagascar; c CIRAD, UMR PVBMT, Saint-Pierre, Reunion Island, France; d INRAE, UMR PVBMT, Saint-Pierre, Reunion Island, France; University of Tennessee at Knoxville

**Keywords:** Madagascar, *Ralstonia pseudosolanacearum*, bacteriocins, molecular epidemiology

## Abstract

Bacterial wilt caused by the Ralstonia solanacearum species complex (RSSC) is a major threat to vegetable crops in Madagascar. For more effective disease management, surveys were carried out in the main vegetable production areas of the country, leading to the collection of 401 new RSSC isolates. Phylogenetic assignment of the isolates revealed a high prevalence of phylotype I sequevar 18. This result contrasts sharply with the epidemiological pattern of RSSC in neighboring islands, including Reunion Island, Comoros, Mayotte, Mauritius, Rodrigues, and the Seychelles, where phylotype I sequevar 31 is widespread. Molecular typing characterization of the Malagasy isolates allowed the identification of 96 haplotypes. Some are found in various plots located in different provinces, which suggests that they were probably disseminated via infected plant material. To find out a potential explanation for the observed epidemiological pattern, we examined the capacity of the Malagasy strains to produce bacteriocin. Interestingly, the highly prevalent genetic lineages I-18 produce bacteriocins that are active against all the genetic lineages present in the country. This work sheds light on the potential impact of bacteriocins in the epidemiology of Malagasy RSSC.

**IMPORTANCE** Knowledge of the epidemiology of a plant pathogen is essential to develop effective control strategies. This study focuses on the epidemiological pattern of Ralstonia pseudosolanacearum phylotype I populations responsible for bacterial wilt in Madagascar. We identified, with the newly collected isolates in three provinces, four genetic lineages probably propagated via infected plant material in Madagascar. We revealed that the epidemiological situation in Madagascar contrasts with that of neighboring Indian Ocean islands. Interestingly, our study on the bacteriocin-producing capacity of Malagasy isolates revealed a correlation between the inhibitory activity of the producing strains and the observed epidemiology. These results suggested that the epidemiology of plant pathogens may be impacted by bacteriocin production.

## INTRODUCTION

To develop effective control strategies against plant pathogens, knowledge of their populations’ genetic diversity and dynamic structure is essential (1 to 6). A fine identification of the genetic lineages responsible for an epidemic is fundamental for disease management. Phenotypic methods based on the characterization of strains according to their expressed traits have been used for years. For example, bacterial strains can be differentiated according to the morphology of their colonies on medium culture, biochemical and serological tests, and their sensitivity or resistance to antibiotics (7). However, the main disadvantage of these typing methods is that they fail to discriminate sufficiently between related strains (7). The advent of genotyping has revolutionized typing methods, with the development of molecular tools that are based on stable, highly discriminative, and reproducible markers (8). Multilocus variable number of tandem repeat (VNTR) analysis (MLVA) has proven to be one of the most valuable genotyping tools for outbreak analysis and for the epidemiological surveillance of phytopathogenic bacteria such as Clavibacter michiganensis subsp. *michiganensis* (9), Pseudomonas syringae pv. *tomato* and *maculicola* (10), Xylella fastidiosa (11), Xanthomonas citri pv. *citri* (1), Xanthomonas vasicola pv. *musacearum* (5), and the Ralstonia solanacearum species complex (6, 12).

However, a fine description of the epidemiological situation does not suffice to control an outbreak. Understanding the factors and mechanisms involved in the epidemic is also important when it comes to implementing relevant disease control strategies. It is assumed that a genetic lineage capable of surviving and proliferating successfully in a competitive environment is likely to thrive in its niche. Several weapons are used by bacteria to prosper in their environment, e.g., the secretion of antimicrobial compounds called bacteriocins, which are ribosomally synthesized proteinaceous compounds active against closely related strains (13). Several studies have highlighted how bacteriocins influence the competitivity and prevalence of a genetic lineage (14 to 18). For instance, in Florida, it was demonstrated that race T3 of Xanthomonas euvesicatoria supplanted race T1 in tomato fields due to its ability to produce bacteriocins (18).

The Ralstonia solanacearum species complex (RSSC) comprises globally distributed plant-pathogenic bacteria that cause bacterial wilt (BW) in 54 botanical families (19). RSSC is considered a major threat to agriculture, more particularly in food-insecure developing countries (20). This species complex is composed of three species, which can be subdivided into four phylotypes according to the strains’ geographical origin. *R. pseudosolanacearum* comprises phylotypes I and III, originating from Asia and Africa, respectively; R. solanacearum includes phylotypes IIA and IIB from America; and *R. syzygii* includes phylotype IV from Indonesia, Japan, and Australia (21 to 23). Furthermore, each phylotype can be subdivided into sequevars, which group together isolates whose partial endoglucanase (*egl*) gene sequence diverges by less than 1% (21). The R. solanacearum species is considered a quarantine pathogen in the European Union (24) and a select agent in the United States (25). BW is known to be one of the most devastating diseases in solanaceous crops in the South-West Indian Ocean (SWIO) islands (Madagascar, Reunion Island, Mauritius, Rodrigues, Mayotte, Comoros, and the Seychelles). Understanding the genetic diversity of the RSSC populations is a prerequisite to the development of suitable BW control strategies in the SWIO. Between 2014 and 2016, a wide sampling survey was conducted in the small SWIO islands (Comoros, Mauritius, Reunion Island, Rodrigues, and the Seychelles). Genetic diversity analysis and the phylogenetic assignment of the isolates highlighted the prevalence of the *R. pseudosolanacearum* phylotype I sequevar 31 (3). In 2020, a newly developed MLVA scheme, specific to phylotype I (RS1-MLVA14), allowed the genotyping of 148 Malagasy isolates from the central highlands (1,000 to 1,600 m) and a lowland region (6 to 50 m). The phylogenetic assignment of the isolates revealed the prevalence of phylotype I sequevar 18. Genetic diversity analysis showed that diversity contrasted significantly according to elevation, with more diverse strains at low altitudes (6). However, a more exhaustive study covering diverse crop production zones at middle and low altitudes was deemed necessary for a more in-depth analysis of the genetic diversity and structure of RSSC populations in Madagascar.

Bacteriocin production by RSSC strains was first reported by Okabe (26). Several studies have partially characterized the bacteriocins as nonsedimentable and belonging to a low molecular weight group (27, 28) or, on the contrary, as sedimentable, thermolabile, trypsin resistant, and belonging to a high molecular weight group (29). In 1996, bacteriocin profiles of RSSC strains from the French West Indies revealed a complete correlation with their group membership (30). In addition, bacteriocin activity was also demonstrated *in planta*, which could confer a selective advantage for the growth of producing strains (30). In 2015, there were suggestions that bacteriocin production may influence the geographical distribution of phylotype IIB sequevar 1 (IIB-1), represented by strain UW551. Indeed, the authors demonstrated that the tropical strain GMI1000 (I-18) and the subtropical strain K60 (IIA-7) produce bacteriocins that inhibit the growth of UW551 at warm temperatures (31). In Madagascar, the bacteriocin-producing capacity of RSSC isolates has not yet been studied.

In this work, we analyzed the genetic diversity and population structure of 401 RSSC isolates, recently collected from various crop production areas in Madagascar. We then conducted the first-ever tests on the bacteriocin activity of isolates that are representative of the RSSC genetic lineages found in Madagascar, in order to determine whether bacteriocin production might influence their epidemiological patterns in this country.

## RESULTS

### Phylogenetic assignments of the Malagasy isolates.

In 2018 and 2019, widespread sampling was conducted in 3 provinces in Madagascar: Mahajanga (3 regions, 11 plots, located at low elevation, ranging from 8 to 49 m), Toamasina (3 regions, 18 plots, located at low and mid elevations, ranging from 5.2 to 792 m), and Antananarivo (3 regions, 6 plots, located at high elevation, ranging from 902 to 1,503 m) (Fig. S1 in the supplemental material). As a result, 401 isolates were collected and called the C2 collection (Table S1). Its phylogenetic assignment revealed the presence of 3 phylotypes in the areas sampled. Moreover, data from a previous work on Malagasy phylotype I strains (C1 collection) (6) were added to the analysis since they include isolates mainly collected in high elevation. One hundred twenty-three strains are from Antananarivo (2 regions, 13 plots, elevation ranging from 1,176.6 to 1,484 m), and 25 strains are from Toamasina (1 region, 5 plots, elevation ranging from 8 to 74.6 m). Combined together (C1+C2), the phylotype I (*n* = 491) represented 89.44% of the Malagasy isolates. It was widely distributed in the provinces of Antananarivo (*n* = 125), Mahajanga (*n* = 252), and Toamasina (*n* = 112), representing 8 sampling regions and 40 plots ranging from sea level to an altitude of 1,484 m. Phylotype I was isolated from a wide range of plant hosts representing 8 botanical families: Solanaceae (*n* = 466), Cucurbitaceae (*n* = 2), Araceae (*n* = 2), Musaceae, (*n* = 1), Fabaceae (*n* = 3), Brassicaceae (*n* = 1), Asteraceae (*n* = 3), Mimosoideae (*n* = 2), and unreported plant hosts (*n* = 11). Phylotype II (*n* = 53) represented 9.65% of the isolates. These were sampled in the Antananarivo and Toamasina provinces, representing 4 regions and 12 plots, located at mid and high elevations from 764 m to 1,503 m. Phylotype II (*n* = 53) was collected from Solanaceae, mostly Solanum tuberosum (*n* = 48). Phylotype III (*n* = 5) represented 0.91% of the isolates. These were collected in Toamasina and Antananarivo, representing 2 regions and 2 plots, located at mid and high elevations from 787 m to 1,240 m. The phylotype III isolates were sampled from Fabaceae (Phaseolus vulgaris; *n* = 1) and Solanaceae (Capsicum annuum; *n* = 4).

The analysis of the endoglucanase (*egl*) gene of 360 isolates (C1+C2), representative of the sampling areas, made it possible to classify them into sequevars. Four sequevars belonging to phylotype I were identified: sequevar 18 (*n* = 276) represented 76.67% of the isolates, sequevar 46 (*n* = 41) represented 11.39% of the isolates, sequevar 33 (*n* = 5) represented 1.39% of the isolates and sequevar 31 were represented by only two isolates. Sequevar 1 belonging to phylotype II represented 8.61% of the isolates (*n* = 31). Sequevar 19 was represented by only one isolate, and sequevar 60 represented 1.11% of the isolates (*n* = 4), both belonging to phylotype III.

### Genetic diversity of the Malagasy phylotype I isolates.

A total of 475 phylotype I isolates were genotyped with the RS1-MLVA14 scheme: 148 isolates from a previous study (C1) (6) and 343 in this study (C2). Overall, 441 isolates were genotyped with all 14 markers, and 34 isolates were genotyped with only 13 markers, despite several assays. Nonetheless, the latter were retained for the genetic diversity analysis. A further 34 isolates were removed from the analysis because they did not amplify on more than one locus.

A total of 96 haplotypes were identified, with the high prevalence of the two haplotypes MT004 (17.47%) and MT007 (17.05%). The Malagasy collections revealed an overall genetic diversity of Hnb = 0.39, varying from 0.18 to 0.48 for Antananarivo and Toamasina provinces, respectively (Table 1). Toamasina Province has the highest allelic richness (A = 2.67), and Antananarivo has the lowest (A = 1.83) (Table 1). The major haplotypes found in Antananarivo and Mahajanga are MT004 and MT007, whereas MT014 and MT334 were the most frequent in Toamasina (Table 1).

**TABLE 1:** Genetic diversity estimated from RS1-MLVA14 data for C1 and C2 RSSC collections^*a*^

Population	Elevation	*n*	A	G	Hnb	Major haplotypes
Provinces						
Toamasina	5.2 to 792 m	99	2.67	42	0.48	MT014 (*n* = 8)
						MT334 (*n* = 6)
Antananarivo	1176.6 to 1484 m	125	1.83	22	0.18	MT004 (*n* = 23)
						MT007 (*n* = 43)
Mahajanga	8 to 49 m	250	2.15	46	0.36	MT004 (*n* = 58)
						MT007 (*n* = 37)
Hosts						
*Solanum lycopersicum*		207	2.04	37	0.28	MT004 (*n* = 60)
						MT007 (*n* = 32)
*Capsicum annuum*		41	2.57	17	0.45	MT003 (*n* = 4)
						MT334 (*n* = 6)
*Solanum melongena*		46	2.48	20	0.38	MT236 (*n* = 14)
						MT266 (*n* = 4)
						MT249 (*n* = 4)
Solanum tuberosum		20	2.31	8	0.34	MT007 (*n* = 6)
						MT023 (*n* = 6)
*Solanum aethiopicum*		99	1.98	18	0.22	MT007 (*n* = 47)
						MT004 (*n* = 18)

a *n* = number of isolates; A = allelic richness calculated by rarefaction method; G = number of haplotypes; Hnb = Nei’s unbiased estimates of genetic diversity.

Three populations were defined according to the Malagasy provinces: Mahajanga (*n* = 250), Antananarivo (*n* = 125), and Toamasina (*n* = 99). Genetic differentiations among the three populations are important and significant: Fst = 0.19***, Rst = 0.24*** between Mahajanga and Antananarivo; Fst = 0.28***, Rst = 0.19*** between Toamasina and Antananarivo; and Fst = 0.19***, Rst = 0.29*** between Toamasina and Mahajanga (Table S3). Nei’s unbiased estimates of genetic diversity (Hnb) ranged from 0.18 to 0.48, and allelic richness varied from 1.83 to 2.67 for Antananarivo and Toamasina, respectively (Table 1).

We defined five populations according to their major plant hosts. They were composed of isolates collected from Solanum lycopersicum (*n* = 207), Solanum melongena (*n* = 46), Capsicum annuum (*n* = 41), Solanum tuberosum (*n* = 20), and Solanum aethiopicum (*n* = 99). *Capsicum annuum* showed the highest diversity (Hnb = 0.45; allelic richness = 2.57). and *Solanum aethiopicum* the lowest diversity (Hnb = 0.22; allelic richness = 1.98) (Table 1). It is worth noting that most isolates collected from *Capsicum annuum* are from Toamasina Province (*n* = 38 out of 41 isolates) (Table 1). The genetic differentiation between *Solanum aethiopicum* and *Solanum lycopersicum* is low (Fst = 0.02*, Rst = 0.00015*). It is moderate but significant between Solanum tuberosum and *Capsicum annuum* (Fst = 0.08*, Rst = 0.007***). It is important and significant between Solanum tuberosum and *Solanum lycopersicum* (Fst = 0.13***, Rst = 0.29***), *Capsicum annuum* and *Solanum lycopersicum* (Fst = 0.16***, Rst = 0.26***), *Solanum melongena* and *Solanum lycopersicum* (Fst = 0.32***, Rst = 0.26***), *Solanum melongena* and *Capsicum annuum* (Fst = 0.26***, Rst = 0.12***), *Solanum aethiopicum* and *Capsicum annuum* (Fst = 0.19***, Rst = 0.16***), Solanum tuberosum and *Solanum melongena* (Fst = 0.34***, Rst = 0.28***), *Solanum aethiopicum* and *Solanum melongena* (Fst = 0.4***, Rst = 0.27***), and *Solanum aethiopicum* and Solanum tuberosum (Fst = 0.16***, Rst = 0.33***) (Table S4).

### Population structure analysis of the Malagasy phylotype I isolates.

**FIG 1:**
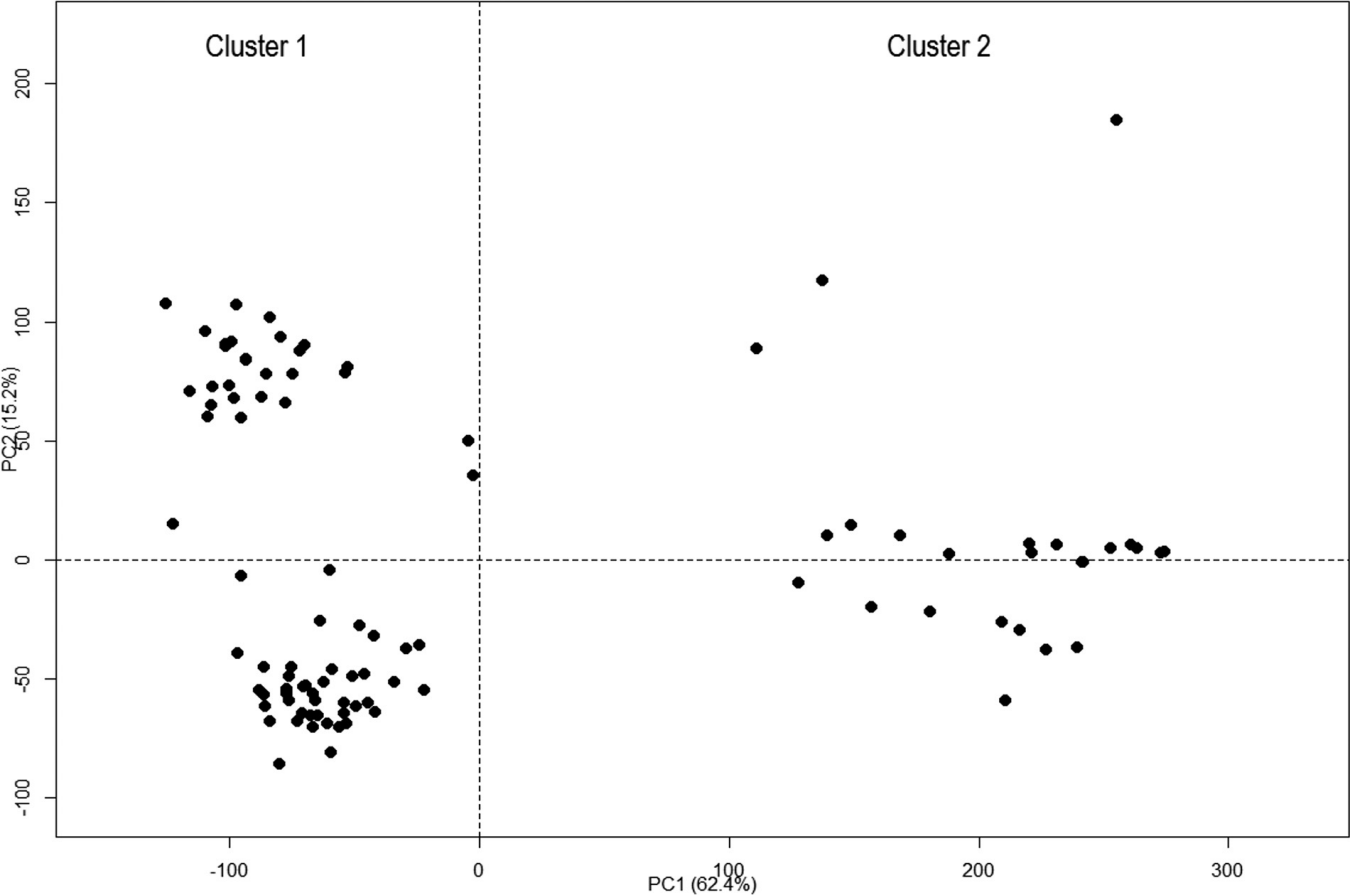
Multidimensional scaling (MDS) of the 96 Malagasy phylotype I haplotypes suggests two clusters with a probability of 52.08%. Cluster 1 groups together the majority of the haplotypes (G = 72), while cluster 2 comprises 24 haplotypes.

A multidimensional scale (MDS) analysis was performed to identify a possible clustering of the 96 Malagasy haplotypes. The MDS plot suggested two clusters with a probability of 52.08%. Haplotypes were assigned to these clusters with a probability of 77.57%. Axis 1 and 2 explained 62.4% and 15.2% of the variance, respectively. Cluster 1 groups together 72 haplotypes, while cluster 2 comprises 24 haplotypes (Fig. 1).

**FIG 2:**
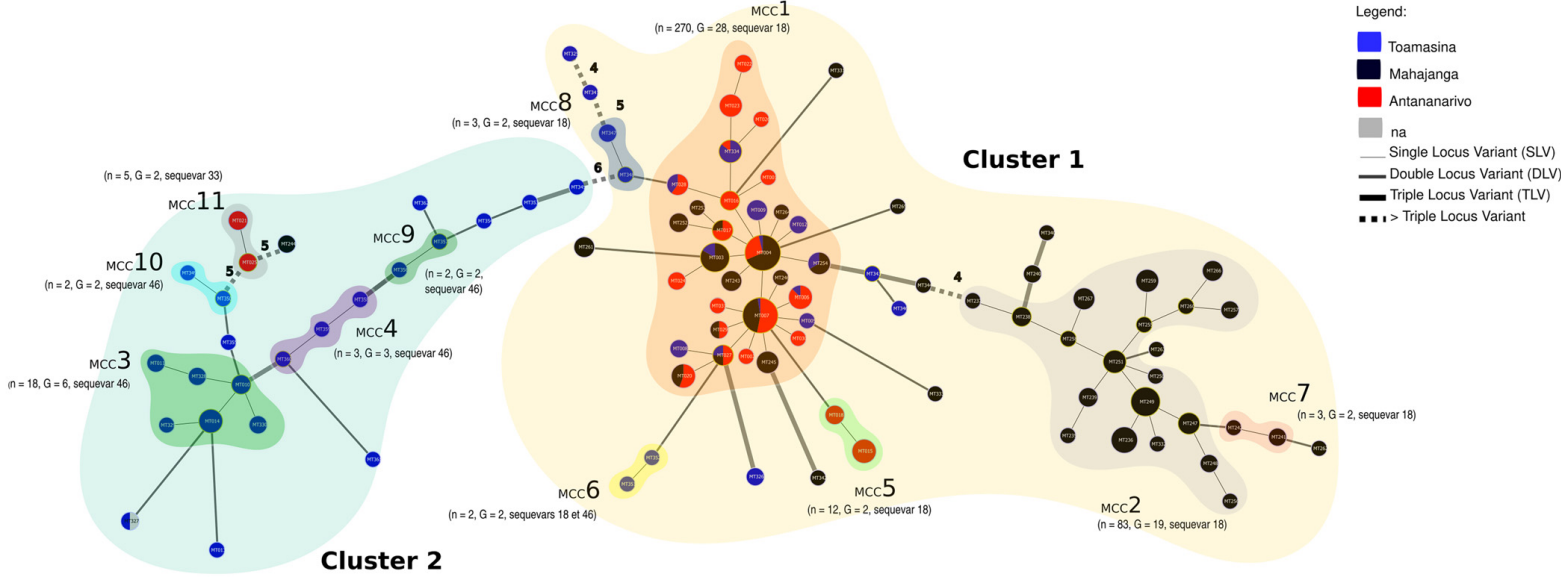
Minimum spanning tree (MST) of the Malagasy phylotype I shows that, according to the province of collection, the haplotypes are distributed among 11 clonal complexes. The haplotypes were identified by using goeBURST full MST in PHYLOVIZ. Each MLVA type (MT) is displayed as a circle, the size of which is proportional to the number of isolates represented. The different colors indicate the province where samples were collected. The branch thickness depends on the number of locus differences between the neighboring haplotypes. MCC1 to MCC11 represent the Malagasy clonal complexes 1 to 11. A clonal complex is composed of haplotypes that differ only by one VNTR locus. Haplotypes were regrouped into two clusters according to the MDS result. Here, *n* represents the number of strains and G represents the number of haplotypes.

A minimum spanning tree (MST) was built to visualize the genetic links between the Malagasy haplotypes. According to the structure of the MST, 11 Malagasy clonal complexes (MCC) were identified (Fig. 2). The major clonal complex MCC1 (*n* = 270) regroups 28 haplotypes belonging to sequevar 18, sampled from the three provinces (Antananarivo, Mahajanga, and Toamasina). The second major clonal complex (MCC2) (*n* = 83) regroups 19 haplotypes belonging to sequevar 18, sampled from Mahajanga Province. The third major clonal complex (MCC3) (*n* = 18) regroups 6 haplotypes belonging to sequevar 46, sampled from Toamasina Province. The 9 remaining clonal complexes (MCC4 to MCC11) are minor, grouping together two to three strains sampled from the three provinces. The MDS analysis shows that cluster 1 includes the majority of strains (*n* = 373, G = 72). In cluster 1, we observed MCC1, MCC2, MCC5, MCC6, MCC7, and MCC8 belonging to sequevar 18, except one isolate from MCC6, identified as sequevar 46. Cluster 2, which regroups 120 isolates and 24 haplotypes, is composed of MCC3, MCC4, MCC9, and MCC10 belonging to sequevar 46, and MCC11 belonging to sequevar 33.

**FIG 3:**
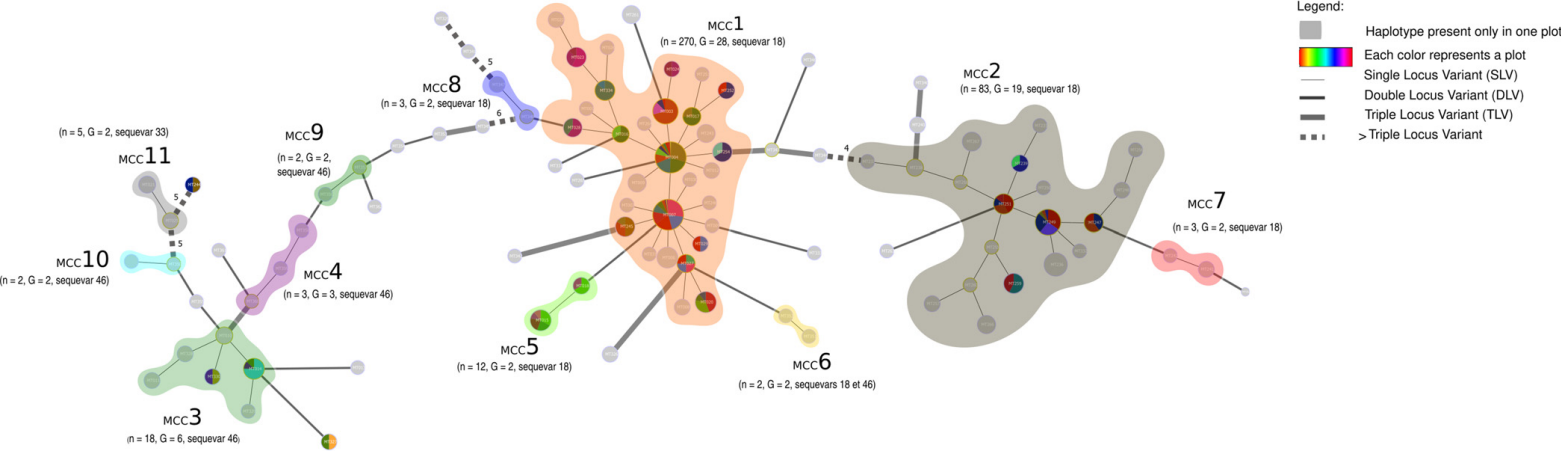
Minimum spanning tree (MST) of the Malagasy phylotype I shows that numerous haplotypes are shared in different plots suggesting dissemination of these haplotypes via infected plant material. The haplotypes were identified by using goeBURST full MST in PHYLOVIZ. Each MLVA type (MT) is displayed as a circle, the size of which is proportional to the number of isolates represented. The different colors indicate the sampling plot. The branch thickness depends on the number of locus differences between the neighboring haplotypes. MCC1 to MCC11 represent the Malagasy clonal complexes 1 to 11. A clonal complex is composed of haplotypes that differ only by one VNTR locus. Here, *n* represents the number of strains and G represents the number of haplotypes.

We found some haplotypes in different plots located in the same province (Fig. 3). For instance, MT014 and MT330, belonging to MCC3, are found in Toamasina; MT015 and MT018, belonging to MCC5, are found in Antananarivo (Fig. 2 and 3). Other haplotypes belonging to the same MCC are found in different plots in various provinces. For example, in MCC1, MT017, MT020, MT029 are found in Mahajanga and Antananarivo; MT003 and MT254 are present in Mahajanga and Toamasina; MT028 and MT334 are in Toamasina and Antananarivo; and MT004, MT007, and MT027 are in Mahajanga, Toamasina, and Antananarivo.

An MST based on the strain samples’ host was also built. It did not reveal a link between host plants and bacterial population structure. Indeed, each clonal complex includes haplotypes isolated from various plants (Fig. S2).

### Genetic links between the Malagasy and worldwide phylotype I isolates.

**FIG 4:**
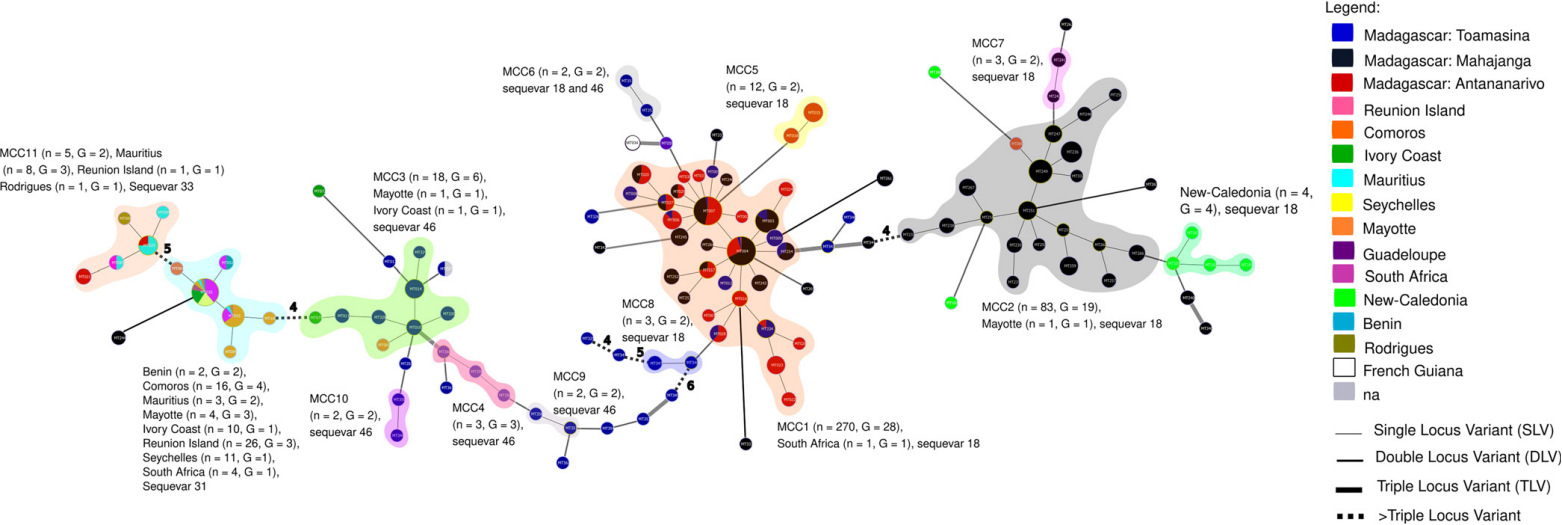
Minimum spanning tree (MST) shows the genetic links between the Malagasy and worldwide phylotype I predominantly from the South-West Indian Ocean and Africa. The haplotypes were identified by using goeBURST full MST in PHYLOVIZ. Each MLVA type (MT) is displayed as a circle, the size of which is proportional to the number of isolates represented. The different colors indicate the sampling country. The reference strain GMI1000 isolated from French Guiana corresponds to the MT034 haplotype. The branch thickness depends on the number of locus differences between the neighboring haplotypes. MCC1 to MCC11 represent the Malagasy clonal complexes 1 to 11. A clonal complex is composed of haplotypes that differ only by one VNTR locus. Here, *n* represents the number of strains and G represents the number of haplotypes.

A global MST was built with the 475 Malagasy isolates and 165 worldwide isolates from the Indian Ocean (*n* = 83), Africa (*n* = 30), the Americas (*n* = 16), Asia (*n* = 17), Australia (*n* = 1), the Pacific Islands (*n* = 6), and the West Indies (*n* = 12). The worldwide isolates that are close to the Malagasy strains are represented in Fig. 4.

Surprisingly, the main Malagasy clonal complex MCC1 (*n* = 270) seems specific to Madagascar. Only the major haplotype MT004 is shared with an isolate sampled in South Africa. The MCC2 forms a clonal complex with MT080 from Mayotte, and MCC3 forms a clonal complex with MT074 from the Ivory Coast and MT081 from Mayotte. As expected, the haplotype MT244 (sequevar 31) is close (double locus variant) to a clonal complex combining sequevar 31 isolates, sampled from the South-West Indian Ocean and Africa. MCC11 forms a clonal complex with haplotypes sampled from Mauritius (MT025, MT085, MT094), Reunion Island (MT085), and Rodrigues (MT098). The minor Malagasy clonal complexes (MCC4, MCC5, MCC6, MCC7, MCC9, MCC10) appear to be specific to Madagascar. The RSSC reference strain isolated from French Guiana, GMI1000 (MT034), differs at three loci from the MT059 sampled in Toamasina (Madagascar).

### Growth inhibition activity.

In this study, we hypothesized that bacteriocin production may be correlated with the prevalence of RSSC genetic lineages in Madagascar. Bacterial supernatant growth inhibition assays were then performed to assess the inhibition activity of 18 isolates that are representative of the 4 genetic lineages found in Madagascar, namely, sequevars 18 (*n* = 11), 31 (*n* = 1), 33 (*n* = 2), and 46 (*n* = 4), against 27 target isolates representative of sequevars 18 (*n* = 19), 31 (*n* = 2), 33 (*n* = 2), and 46 (*n* = 4).

**FIG 5:**
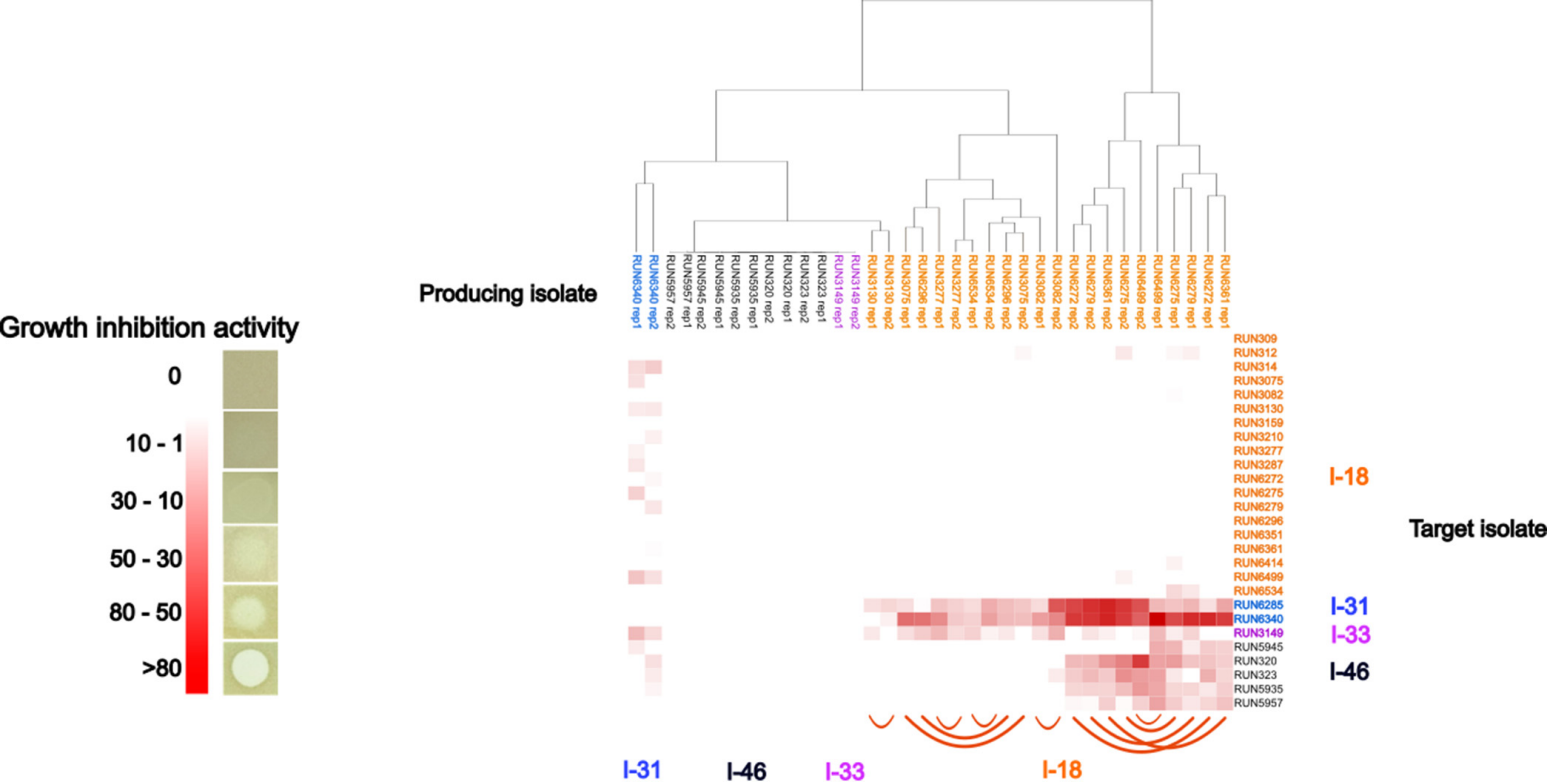
Inhibitory activity of 18 bacteriocin-producing Malagasy isolates on 27 target isolates shows that the highly prevalent genetic lineage I-18 produces bacteriocins that are active against all other phylotype I genetic lineages present in Madagascar. The dendrogram was based on the similarity/dissimilarity of the inhibitory activity patterns of the 18 producing isolates. Links between the different patterns are colored according to the sequevar of the isolates (I-18, I-31, I-33, and I-46). The heatmap was based on the target isolates’ sensitivity to the antimicrobial compounds. The target isolates were grouped according to their sequevar.

According to their patterns of growth inhibition activity, isolates from sequevar 18 were classified into two clusters (Fig. 5; Fig. S3). The first cluster regroups five isolates belonging to the second major Malagasy clonal complex (MCC2, *n* = 3) and its closely related clonal complex (MCC7, *n* = 2). They were collected in the province of Mahajanga, regions of Boeny (*n* = 3) and Sofia (*n* = 2). They produce an antimicrobial substance that is highly active against the two sequevar 31 isolates identified in Madagascar and collected in Mahajanga Province, in the regions of Betsiboka (RUN6285) and Sofia (RUN6340). These sequevar 18 supernatants are also moderately active against strains belonging to sequevars 33 and 46 and are even weakly active against other sequevar 18 isolates. The second cluster regroups six isolates belonging to the major Malagasy clonal complex (MCC1, *n* = 4), its closely related clonal complex (MCC5, *n* = 2), and an isolate belonging to the MCC2 (RUN3130). They were collected in the provinces of Antananarivo (Itasy and Analamanga regions, *n* = 4), Toamasina (Alaotra-Mangoro region, *n* = 1), and Mahajanga (Sofia region, *n* = 1). Their supernatants are weakly to moderately active against sequevar 31 isolates and one sequevar 33 isolate. Regarding the remaining sequevars, supernatants from sequevars 33 and 46 present no growth inhibition activity against all the isolates tested, whereas the supernatant from sequevar 31 (RUN6340 strain) is weakly to moderately active against a wide range of isolates belonging to sequevars 18, 33, and 46 (Fig. 5).

Partial characterization of all the supernatants revealed that their inhibitory activities are sensitive to high temperature (100°C, 10 min). They were degraded by proteinase K at a final concentration of 50 μg/mL, except for those from the MCC2 (*n* = 3) and MCC7 (*n* = 2), and were only active against RSSC isolates since non-RSSC strains (Table S7) were resistant to RSSC bacteriocins. When subject to serial dilution, a clearing zone became uniformly more turbid with greater dilution. This is characteristic of supernatants containing bacteriocins, except for RUN3130, which resulted in individual plaques, probably due to bacteriophages.

## DISCUSSION

### High prevalence of the phylotype I sequevar 18 in Madagascar.

Overall, the genetic analysis of Malagasy RSSC isolates collected in 2018 and 2019 confirmed the analysis of Malagasy RSSC isolates collected in 2006 to 2013 (C1) (6). It also provides new information because research was extended to 6 new regions in the provinces of Antananarivo, Toamasina, and Mahajanga. This revealed that RSSC is well established in Madagascar with the presence of three phylotypes (I, II, and III), with a high prevalence of phylotype I in the country, representing 89.44% of the isolates. Interestingly, the Malagasy phylotype I isolates showed greater genetic diversity in the lowlands (provinces of Mahajanga and Toamasina), where there are seaports, than in the highlands (province of Antananarivo). Mahajanga’s seaport is mainly used for local traffic along Madagascar's west coast and to neighboring islands (primarily the Comoros and Mayotte). Toamasina’s seaport is the nation's main port and is used for national and international traffic. The trade of plant products is known to play a major role in pest and disease spread (32). For example, recently, exchanges of infected *Rosa* cuttings were probably responsible for the introduction of phylotype I strains in *Rosa* cut flower production units in Europe (33). We can assume that these seaports are points of entry/exit for contaminated plant products and therefore contribute to the great genetic diversity in these provinces and to the dispersion of RSSC strains to other provinces. Interestingly, several phylotype I isolates were present in the highlands, in Antananarivo Province, which is remarkable because until now, reports suggest that phylotype I strains were not well adapted to cool temperatures (6).

Sixty-six new haplotypes were characterized during this study. They are distributed among 11 clonal complexes, seven of which were new discoveries compared to our previous work in Madagascar (6), Moreover, our study on the strains’ geographical distribution revealed that many haplotypes were shared between different plots located in the three provinces of Antananarivo, Toamasina, and Mahajanga. These observations strongly suggest that the transmission of these haplotypes from one plot to another occurs via contaminated plant material, including seeds, since access to certified seeds remains limited in Madagascar.

Phylotype IIB-1 strains, unlike phylotype III strains, are known to be spread by seed potato tubers throughout the world (4, 34). However, the transmission of RSSC via true seeds is still a matter of debate, although a few studies have reported its possible transmission via tomato seeds (35, 36), eggplant seeds (37 to 40), and chilli seeds (41). Hence, it is important to conduct studies on the transmission of Malagasy strains via solanaceous seeds. No distinct population structure was observed in correlation to the host plant origin of the Malagasy haplotypes, confirming previous reports (4, 6), which strongly suggests that each phylotype I haplotype has the capacity to infect a wide range of hosts.

Our analysis showed genetic links between the Malagasy and worldwide strains, predominantly from the SWIO and Africa. This is the case for the major Malagasy clonal complexes: MCC1 (sequevar 18), MCC2 (sequevar 18), MCC3 (sequevar 46), and for the minor Malagasy clonal complex MCC11 (sequevar 33). All these genetic links strongly suggest exchanges of infected plant material among Madagascar, the SWIO islands, and Africa (www.tresor.economie.gouv.fr/se/madagascar/, www.lexpress.mu/) (42 to 44).

Phylogenetic assignment based on the partial *egl* gene sequence allowed the identification of four phylotype I sequevars. Sequevar 18 was the most widespread (76.67%), followed by sequevars 46 (11.39%), 33 (1.39%), and 31 (only two isolates). This result is surprising as sequevar 31 is the most prevalent in the small SWIO islands (3). The two I-31 isolates, detected in Mahajanga Province, were probably introduced in Madagascar via the seaport because of intense traffic with neighboring islands, especially the Comoros and Mayotte, where sequevar 31 was reported to be highly prevalent (3, 45).

Another interesting point of this study is that we confirmed that the RS1-MLVA14 scheme globally retains the phylogenetic signal (6), since our MST analysis showed that the isolates were grouped according to their phylogenetic assignment (sequevar). This is important as it is known that some phylotype I sequevars are polyphyletic and thus the sequevar system is not completely robust to reflect phylogenetic relationships for phylotype I strains (46, 47). The RS1-MLVA14 scheme could be an alternative to the sequevar system to classify within-phylotype I strains, but we revealed one exception in MCC6 that gathers sequevar 18 isolates and one sequevar 46 isolate that could be due to recombination as it is reported that phylotype I is highly recombinogenic (3, 47, 48).

### Broad spectrum inhibition activity of sequevars 18 and 31.

Most microorganisms constantly compete for space and resources (49). To thrive in their environment, bacteria deploy a wide range of mechanisms that harm and kill their competitors (50). One such mechanism involves the production of bacteriocins, ribosomal protein compounds with antimicrobial activity (51). In this study, we revealed *in vitro* direct antagonism of strains representative of the four RSSC genetic lineages identified in Madagascar. Our results suggested the presence of an *in vitro* growth inhibition activity due to bacteriocins (13, 52, 53). Indeed, partial characterization of these growth inhibitors showed that they are only active against RSSC isolates, and their sensitivity to high temperature (100°C) and proteinase K apart from the supernatants of sequevar 18 (MCC2, *n* = 4 and MCC7, *n* = 2). Although proteinase K is a relatively unspecific proteolytic enzyme (a serine protease) (54), some bacteriocins characterized in the literature are also insensitive to proteinase K (55, 56).

We showed that *in vitro* bacteriocin production and bacteriocin sensitivity vary according to genetic lineage. We also demonstrated that some strains of sequevar 18 inhibit strains from the same sequevar. The inhibitory activity assays allowed us to establish a bacteriocin typing scheme corresponding to the status of target strains (resistant or sensitive) as well as bacteriocin-producing strains (high or low capacity). Similar bacteriocin schemes have been highlighted previously for RSSC strains (29, 30, 57). Previous studies revealed that bacteriocins influenced the competitivity and prevalence of a genetic lineage for several bacterial species (14 to 18). Our results suggest the potential impact of bacteriocin production on the geographical distribution and prevalence of RSSC genetic lineages. The less prevalent genetic lineages, sequevars 33 and 46, did not show bacteriocin activity against the representative set of Malagasy strains tested during this study. On the contrary, the most prevalent genetic lineage, sequevar 18, has a broad-spectrum inhibition activity, which may enhance its competitivity and prevalence in its environment. Interestingly, sequevar 18 is globally distributed since it has been isolated in the Americas (58 to 60), Africa (61, 62), the Indian Ocean (3, 45), Asia (63, 64), and Oceania (65), suggesting that this lineage is especially competitive and successful in part due to its bacteriocin-producing capacity. In Madagascar, sequevar 31 also produced antimicrobial compounds active against the genetic lineages present in this country (sequevars 18, 33, and 46). Although sequevar 31 is widely disseminated in the small SWIO islands (3), only two strains were detected in Madagascar. We assume that sequevar 18’s strong antibiosis activity against sequevar 31 could be a determining factor, since other factors may be involved in bacterial competition, such as the type VI secretion system, whereby bacterial cells inject toxins or other molecules into neighboring adversaries to promote cell lysis (66 to 71). Another hypothesis is that sequevar 31 was only recently introduced in Madagascar and has not yet had the time to spread in this country. The plants cultivated in Madagascar might also impact the prevalence of the RSSC genetic lineages. For example, a study revealed that two widely used potato varieties in Madagascar, 720118 (Jaingy) and 800934 (Miova), are susceptible to several Malagasy sequevar 18 strains and resistant to a sequevar 31 strain (12). Interactions between RSSC and phage communities in natural environments could be an alternative explanation of the differential prevalence of RSSC genetic lineages in Madagascar (72).

Since we showed that the susceptibility of strains varies according to their genetic lineages, why are some strains sensitive or immune to particular bacteriocins? The mechanisms of target cell recognition and immunity are yet poorly understood issues in the biology of these antimicrobial peptides (73). Most bacteriocins kill target cells by permeabilization of the cell membrane, and the activity is often very specific, since they employ specific receptors on the target cell surfaces (74). For instance, the β-barrel outer membrane protein insertase, BamA, represents the primary bacteriocin selectivity determinant in pseudomonads (75). Analysis of the L6 surface-exposed loop sequences of BamA in a panel of Pseudomonas aeruginosa strains (*n* = 80) susceptible to pyocins L1 and L2 (76) revealed that pyocins L1 only killed strains sharing the same L6 sequence. Likewise, the subset of pyocin L2-susceptible strains shared a single L6 sequence, different from the one present in pyocin L1-killed strains surface-exposed loops (L6) (75). Furthermore, it has been shown that a set of bacteriocins produced by both Gram-positive and Gram-negative species can employ the membrane components of the mannose phosphotransferase system (Man-PTS) on sensitive cells as receptor molecules (74). A work done on Lactobacillus sakei strains showed a correlation between the Man-PTS gene expression level and the degree of sensitivity to class IIa bacteriocins (77).

This study set out to describe and further our understanding of the epidemiological pattern of the Ralstonia pseudosolanacearum phylotype I in Madagascar. We revealed that sequevar 18 was the most prevalent among the four genetic lineages identified. The bacteriocin typing assays revealed a correlation between the inhibitory activity of the producing strains and the observed epidemiology. This work could lead to further research involving bacteriocin typing assays on all the genetic lineages belonging to phylotypes I, IIB, and III found in the country. As a perspective of this analysis and to find out if the same correlation is observed *in situ*, further studies could be considered to include RSSC isolates collected from other inoculum reservoirs such as soil and irrigation water, in order to compare their genetic diversity and their ability to produce bacteriocins. Moreover, competition assays between these genetic lineages should be performed to establish whether the most prevalent sequevar 18 is also more competitive *in vitro* and *in planta.* Lastly, it would be relevant to extend this work by a comparative genomics study to describe gene content differences between the Malagasy RSSC genetic lineages and to identify those that could explain the RSSC epidemiological patterns in Madagascar.

## MATERIALS AND METHODS

### Bacterial collections.

Three collections of RSSC isolates were used in this study. All isolates were collected from stem fragments of plants belonging to Solanaceae (*n* = 658), Geraniaceae (*n* = 8), Fabaceae (*n* = 8), Araceae (*n* = 5), Cucurbitaceae (*n* = 5), Asteraceae (*n* = 4), Amaranthaceae (*n* = 4), Mimosoideae (*n* = 2), Musaceae (*n* = 2), Brassicaceae (*n* = 2), Zingiberaceae (*n* = 1), Begoniaceae (*n* = 1), Apiaceae (*n* = 1), Casuarinaceae (*n* = 1), Vitaceae (*n* = 1), and unreported plant hosts (*n* = 11) (Tables S1 and S2).

The first two collections (C1 + C2) (Table S1) were collected from three provinces in Madagascar, representing various crop production zones ranging from sea level to an altitude of 1,503 m (Fig. S1). The difference in elevation provided the opportunity to study the influence of altitude on the genetic diversity and structure of RSSC populations. The first collection, C1, was composed of 148 phylotype I isolates, which were isolated in 2006 and 2013 from Toamasina and Antananarivo provinces (6). Toamasina province is represented by the region of Atsinanana (*n* = 25, 4 plots), located in the lowlands (mean elevation = 28.31 m). Antananarivo province is represented by the regions of Analamanga (*n* = 28, 4 plots) and Itasy (*n* = 95, 8 plots), located in the central highlands (mean elevation = 1,057.23 m). The second collection, C2, comprised 401 isolates collected during this study in 2018 and 2019. These isolates originated from the provinces of Toamasina, Mahajanga, and Antananarivo. Toamasina Province is represented by the regions of Atsinanana (*n* = 71, 7 plots), located in the lowlands (mean elevation = 12.61 m), Analanjirofo (*n* = 9, 4 plots), located in the lowlands (mean elevation = 158 m), and Alaotra-Mangoro (*n* = 43, 7 plots), located at mid elevation (mean elevation = 779.42 m). Mahajanga Province is located in the lowlands and is represented by the regions of Betsiboka (*n* = 10, 1 plot, elevation = 44 m), Boeny (*n* = 30, 3 plots, elevation = 12 m), and Sofia (*n* = 212, 7 plots, mean elevation = 41.2 m). Antananarivo Province is located in the central highlands of Madagascar. It is represented by the regions of Analamanga (*n* = 12, 3 plots, mean elevation = 1,294.65 m), Bongolava (*n* = 7, 2 plots, mean elevation = 905.14 m), and Itasy (*n* = 5, 1 plot, mean elevation = 1,503 m). Two isolates are from unreported regions. In order to identify the genetic relationships between the Malagasy and international phylotype I isolates, the analysis included a third collection, C3, corresponding to 165 worldwide isolates from the SWIO islands (*n* = 83), Africa (*n* = 30), Asia (*n* = 17), the Americas (*n* = 16), West Indies (*n* = 12), Pacific Islands (*n* = 6), and Australia (*n* = 1) (Table S2).

### Phylogenetic assignment.

Phylogenetic assignment was performed to verify that the isolates belonged to RSSC and to determine their phylogenetic groups. First, multiplex PCR amplification (21) was performed in 25-μL reaction volumes containing 1× Green GoTaq Reaction Buffer; 1.5 mM MgCl_2_; 0.2 mM dNTP; 2.4 mM of one of the following primers: Nmult21:1F primer specific to phylotype I, Nmult21:2F primer specific to phylotype II, 7.2 mM Nmult23:AF primer specific to phylotype III, Nmult22:InF primer specific to phylotype IV, Nmult22:RR as a reverse primer; 1.6 mM 759F/760R primers specific to RSSC, and 1.25U μL GoTaq flexi DNA polymerase (Promega); 2 μL of DNA suspension; and 10.88 μL sterile HPLC-grade water. PCR was carried out in a Verity 96-well thermal cycler (Applied Biosystems) under the following conditions: an initial denaturation step at 96°C for 5 min, 30 cycles of denaturation at 94°C for 15 s, annealing at 59°C for 15 s, extension at 72°C for 30 s, and a final extension step at 72°C for 10 min. Then, 10 μL of PCR product was loaded into a 1.5% (wt/vol) Sea-Kem1 LE Agarose (Lonza, Basel, Switzerland) gel for electrophoresis. Ethidium bromide was used to stain the gels, and the G:BOX gel imaging system (Syngene, Cambridge, UK) enabled the visualization of the bands under UV light. The molecular weights were estimated by comparison with a 100-bp DNA ladder (Promega, Madison, WI, USA). Second, the partial endoglucanase (*egl*) gene of RSSC isolates was amplified in 50-μL reaction volumes containing 1× Green GoTaq Reaction Buffer, 1.5 mM MgCl_2_, 0.2 mM dNTP, 0.25 μM each of the primers EndoF and EndoR, 1.25U μL GoTaq flexi DNA polymerase (Promega), 2 μL of DNA suspension, and 31.25 μL sterile HPLC-grade water. PCR was performed in a Verity technology thermocycler (Applied Biosystems thermal cyclers) under the following conditions: an initial denaturation step at 96°C for 9 min, 30 cycles of denaturation at 95°C for 1 min, annealing at 70°C for 1 min 30 s, extension at 72°C for 2 min, and a further extension step at 72°C for 10 min. Afterwards, 5 μL of PCR product was separated on 1% agarose gel to visualize the amplification. Positive PCR products were sent to Genewiz (Leipzig, Germany) for DNA double-stranded sequencing (forward and reverse). Forward and reverse chromatograms were analyzed using the Geneious v10.0.9 software (78). Isolates for which the *egl* sequence diverges less than 1% were grouped together to form a sequevar (21).

### MLVA typing.

The objective of the MLVA typing was to determine the allelic profile of the isolates, i.e., the haplotype or multilocus type (MT), which means they can be distinguished at a fine level. This was accomplished by applying the RS1-MLVA14 scheme on phylotype I isolates following the previously described protocol (6). Briefly, a multiplex PCR amplification was carried out in a 15 μL reaction volume containing 7.5 μL Terra PCR Direct Buffer 2× (Terra PCR Direct Polymerase kit, Clontech Laboratories, Inc.), 0.3 μL Terra PCR Direct Polymerase Mix—1.25 U/μL, 1.5 μL 5× Q-solution (Qiagen1, Hilden, Germany), 2.3 μL of a forward and reverse primer mix (2 μM each), 2.4 μL sterile HPLC-grade water, and 1 μL of bacterial suspension as a template. PCR was performed in a Verity 96-well thermal cycler (Applied Biosystems) under the following conditions: an initial denaturation step at 98°C for 2 min, 25 cycles of denaturation at 98°C for 10 s, annealing at 62°C for 15 s, extension at 68°C for 1 min, and a further extension step at 68°C for 30 min. Then, PCR products were diluted (at least 1:80) to avoid peak saturation. In a 2-mL Eppendorf tube, 1,080 μL formamide (Hi-Di formamide, Applied Biosystems) was mixed with 20 μL size marker (GeneScanTM-500 LIZ1 Size Standard, Applied Biosystems). In each well of a 96 well plate, 11 μL was distributed with 1 μL of diluted PCR product. The samples were denatured at 95°C for 5 min, cooled immediately on ice, and loaded onto an ABI Prism 3130XL Genetic Analyzer for capillary electrophoresis.

### Genetic structure and diversity analysis.

The genotyping results were visualized and analyzed with Geneious v10.0.9 (78). They were presented as electropherograms (peaks) of different colors. Each color corresponds to the fragment size of a VNTR locus, which is estimated using the third order least-squares algorithm implemented in Geneious v10.0.9, then a bin size is attributed. The latter takes into account small size variation due to experimental variation. When a VNTR array was truncated, the VNTR number was rounded up to the nearest bin (1, 5, 79). In addition, the number of repeats of VNTR was calculated using the following formula:

$$\mathrm{Number}\;\mathrm{of}\;\mathrm{repeats}\;=\;\frac{\mathrm{Bin}\;\mathrm{size}\;-\;\mathrm{Forward}\;\mathrm{and}\;\mathrm{reverse}\;\mathrm{primer}\;\mathrm{size}\;-\;\mathrm{Flanking}\;\mathrm{region}\;\mathrm{size}}{\mathrm{VNTR}\;\mathrm{size}}$$

The genetic profile of each isolate, i.e., the haplotype or multilocus type (MT), was defined in terms of the number of repeats at each VNTR loci.

The genetic diversity of the Malagasy phylotype I isolates belonging to the C1 and C2 collections was analyzed according to their province of origin and the plant host from which the samples were collected. Arlequin v3.5.2.2 (80) was used to compute Nei’s unbiased estimates of genetic diversity (Hnb). Allelic richness was estimated using HP-RARE (81). A standardized measure of the genetic variance among the populations, Fst and Rst, was also estimated using Arlequin v3.5.2.2 to provide information on the possible gene flow between the Malagasy phylotype I populations.

The presence or absence of genetic structure in the Malagasy phylotype I isolates (C1 + C2) was estimated by computing multidimensional scaling (MDS) with the R package *Bios2mds* (82). MDS is a method that represents measurements of similarity (or dissimilarity) among pairs of objects as distances between points of low-dimensional or multidimensional space (83). Afterwards, the level of differentiation between Malagasy phylotype I isolates (C1 + C2) was represented by minimum spanning trees (MSTs) according to the province where the sample was collected. The MST was also built with the Malagasy (C1 + C2) and worldwide (C3) phylotype I isolates to represent their genetic relationships. All the MSTs were built using global optimal eBURST (goeBURST) and Euclidean distances implemented in Phyloviz (http://www.phyloviz.net/). A clonal complex (CC) was defined as a cluster of haplotypes in which all haplotypes are linked as single locus variants (SLVs) to at least one other haplotype. In this model, the founder haplotype first increases in number before mutation generates SLVs (84).

### Inhibition activity assays.

All the isolates used in this study are representative of the phylotype I genetic lineages found in Madagascar and belonging to the C1 and C2 collections. Direct antagonism of 18 isolates was tested on 27 target isolates using an overlay plate growth inhibition assay, as previously described (52, 85). A log-phase aliquot (25 μL) of each target isolate grown in nutrient broth (approximately 10^9^ CFU/mL) was added to 4.5 mL of molten Kelman soft agar (0.75% [wt/vol] agar) cooled to 45°C and poured over a 2,3,5-triphenyltetrazolium chloride-agar plate (86). The soft agar layer was allowed to solidify, and then 10 μL of filtrate (filter pore size 0.2 μm) of the culture supernatant of each producer isolate (grown in nutrient broth for 24 h) was spotted onto the plate. Plates were incubated at 28°C for 48 h to allow observation of inhibition zones. The experiment was repeated twice to verify the repeatability of the results (Fig. S3).

### Characterization of inhibitory activity in culture supernatants.

Supernatants from the 18 producer isolates used in growth inhibition activity assays were characterized by treating 100 μL at 100°C for 10 min; 50 μL of proteinase K at a final concentration of 50 μg/mL was also added to 50 μL of supernatants and incubated at 37°C for 2 h. After treatment, they were tested for inhibitory activity against a target strain. To differentiate between bacteriophage and bacteriocin inhibition activity, supernatants were serially diluted in water and spotted onto soft agar plates following the protocol described above. When a serial dilution is performed on a supernatant containing bacteriophage, greater dilution results in fewer individual plaques, whereas serial dilution of a supernatant containing bacteriocin results in a clearing zone, which becomes uniformly more turbid with greater dilution (53). The inhibition activity of the supernatants was also tested on 19 strains belonging to bacterial species more or less closely related to RSSC (Table S7): Cupriavidus necator, Ralstonia pickettii, Pseudomonas fluorescens, Pseudomonas savastanoi pv. *savastanoi*, Pseudomonas cichorii, Pseudomonas corrugata, Pseudomonas syringae pv. *tomato*, Pseudomonas putida, Dickeya dadantii, Robbsia andropogonis, Xanthomonas phaseoli pv. *dieffenbachiae*, Xanthomonas vesicatoria, Xanthomonas citri pv. *citri*, Xanthomonas hortorum *pv. gardneri*, Xanthomonas euvesicatoria pv. *euvesicatoria*, Xanthomonas euvesicatoria pv. *perforans*, Clavibacter michiganensis subsp. *michiganensis*, and Escherichia coli.

### Data analysis.

A piece of bacterial lawn in the center of the petri dish was removed manually to serve as a positive control, i.e., a bacteria-free zone, equivalent to 100% bacterial inhibition, in the calculation of inhibitory activity. Data were obtained by placing the plates on a luminous source before photographing them. Image analysis was performed using imageJ software (https://imagej.nih.gov/). The light intensity through the inhibition zones, the bacterial lawn, and the positive control were compared using the following formula:

$$\mathrm{Inhibitory}\;\mathrm{activity}\;=\;\frac{\mathrm{Light}\;\mathrm{intensity}\;\mathrm{through}\;\mathrm{the}\;\mathrm{halo}\;-\;\mathrm{Light}\;\mathrm{intensity}\;\mathrm{through}\;\mathrm{the}\;\mathrm{bacterial}\;\mathrm{lawn}}{\mathrm{Light}\;\mathrm{intensity}\;\mathrm{through}\;\mathrm{the}\;\mathrm{positive}\;\mathrm{control}\;-\;\mathrm{Light}\;\mathrm{intensity}\;\mathrm{through}\;\mathrm{the}\;\mathrm{bacterial}\;\mathrm{lawn}}$$

Two data sets were obtained from the two biological replicates of the overlay plate growth inhibition assay. To verify the repeatability of the results, they were combined and analyzed together. A dendrogram was built on R v4.1.3 to visualize the clustering of similar data based on the Minkowski distance, which is a generalization of both the Euclidean distance and the Manhattan distance. The dendrogram was converted to a Newick format using the phylogram R package and imported to iTOL v6 (https://itol.embl.de) to generate the final tree and heatmap, based on the sensitivity of the target isolates.
