# Supplementary material for: Bacteriocin Production Correlates with Epidemiological Prevalence of Phylotype I Sequevar 18 Ralstonia pseudosolanacearum in Madagascar
Source: Appl Environ Microbiol. 2023 Jan 5;89(1):e01632-22. doi: 10.1128/aem.01632-22 (PMC9888187; doi:10.1128/aem.01632-22)
Supplement: Supplemental file 2 — Supplemental material. Download aem.01632-22-s0002.pdf, PDF file, 0.7 MB [file aem.01632-22-s0002.pdf]

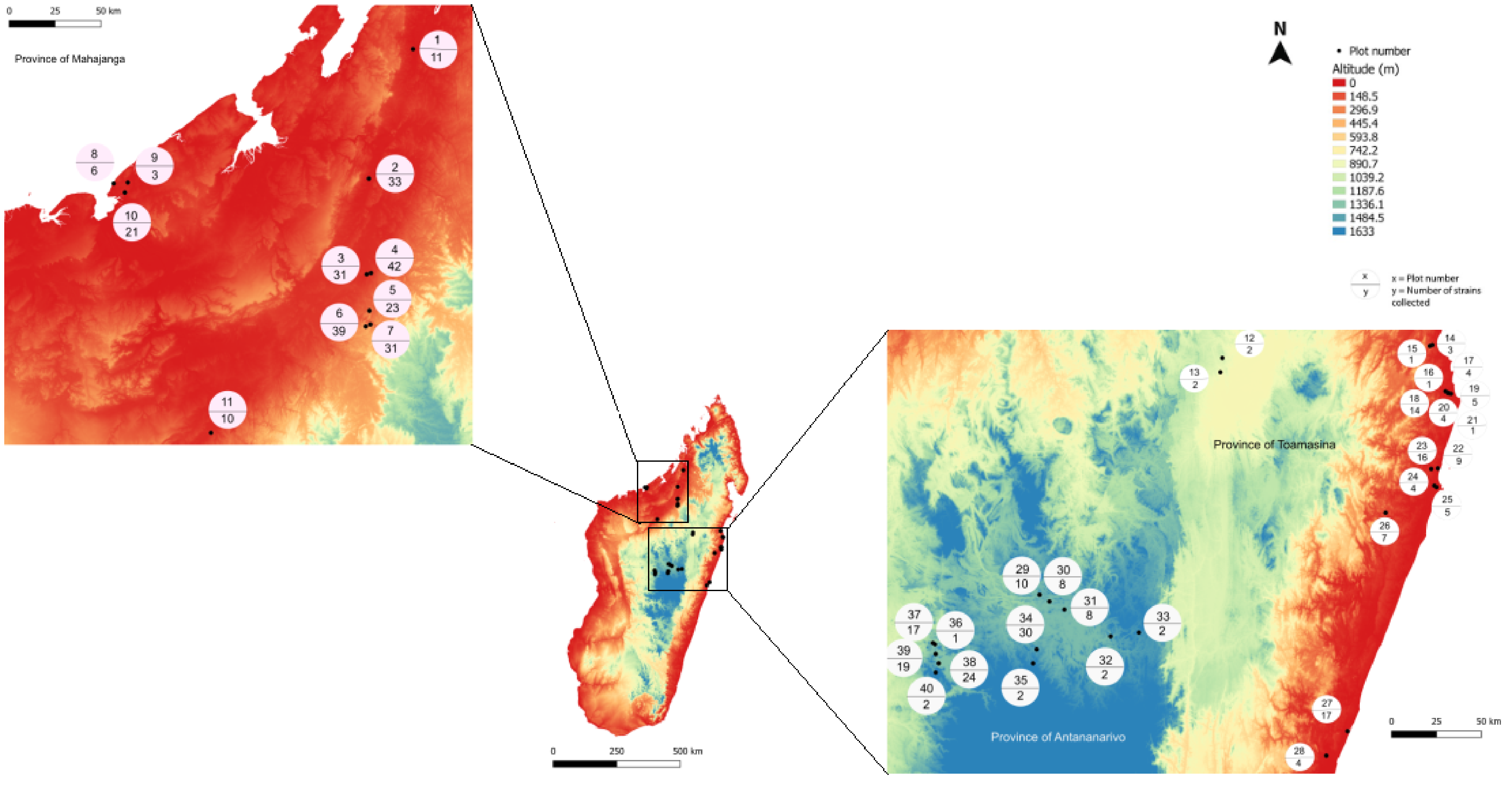
0 25 50 km
Province of Mahajanga
1 11
2 33
3 31
4 42
5 23
6 39
7 31
8 6
9 3
10 21
11 10
N
Plot number
Altitude (m)
0
148.5
296.9
445.4
593.8
742.2
890.7
1039.2
1187.6
1336.1
1484.5
1633
x y
x = Plot number
y = Number of strains collected
0 250 500 km
Province of Toamasina
Province of Antananarivo
12 2
13 2
14 3
15 1
16 1
17 4
18 14
19 5
20 4
21 1
22 9
23 16
24 4
25 5
26 7
27 17
28 4
29 10
30 8
31 8
32 2
33 2
34 30
35 2
36 1
37 17
38 24
39 19
40 2
0 25 50 km

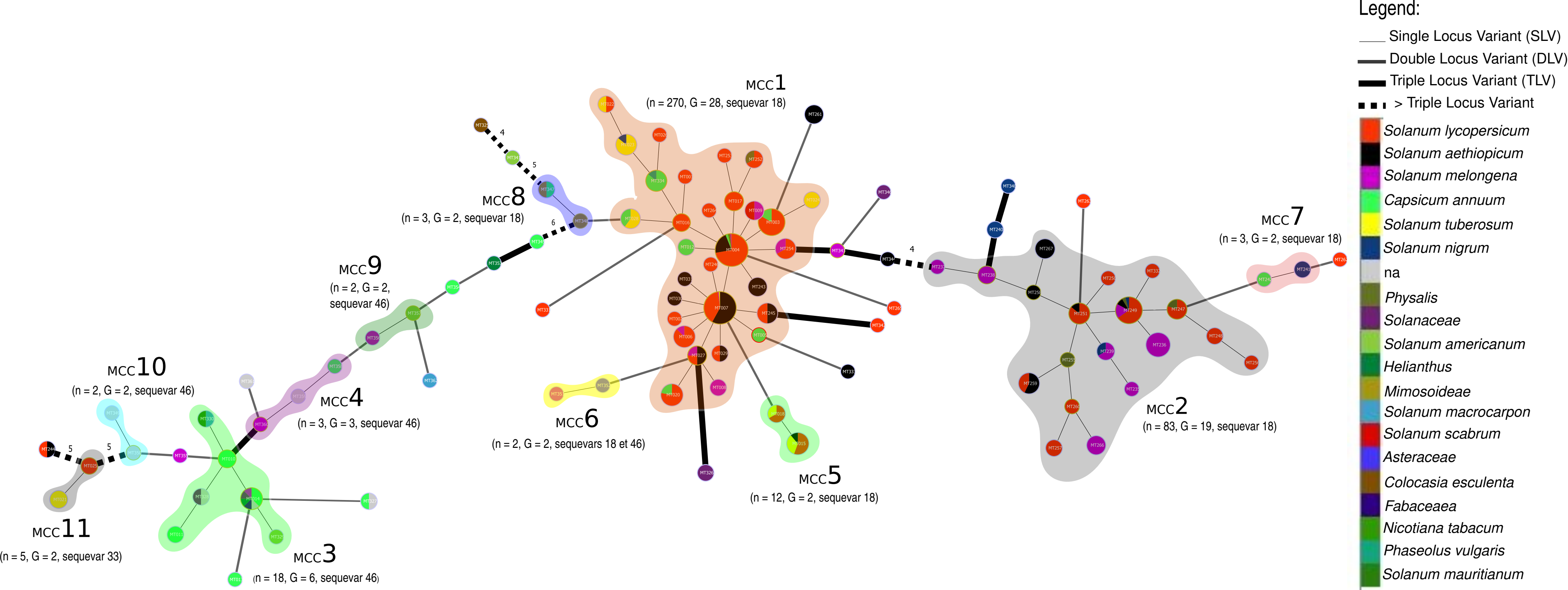
MCC1
(n = 270, G = 28, sequevar 18)
MCC2
(n = 83, G = 19, sequevar 18)
MCC3
(n = 18, G = 6, sequevar 46)
MCC4
(n = 3, G = 3, sequevar 46)
MCC5
(n = 12, G = 2, sequevar 18)
MCC6
(n = 2, G = 2, sequevars 18 et 46)
MCC7
(n = 3, G = 2, sequevar 18)
MCC8
(n = 3, G = 2, sequevar 18)
MCC9
(n = 2, G = 2, sequevar 46)
MCC10
(n = 2, G = 2, sequevar 46)
MCC11
(n = 5, G = 2, sequevar 33)
Legend:
Single Locus Variant (SLV)
Double Locus Variant (DLV)
Triple Locus Variant (TLV)
> Triple Locus Variant
Solanum lycopersicum
Solanum aethiopicum
Solanum melongena
Capsicum annuum
Solanum tuberosum
Solanum nigrum
na
Physalis
Solanaceae
Solanum americanum
Helianthus
Mimosoideae
Solanum macrocarpon
Solanum scabrum
Asteraceae
Colocasia esculenta
Fabaceaea
Nicotiana tabacum
Phaseolus vulgaris
Solanum mauritianum

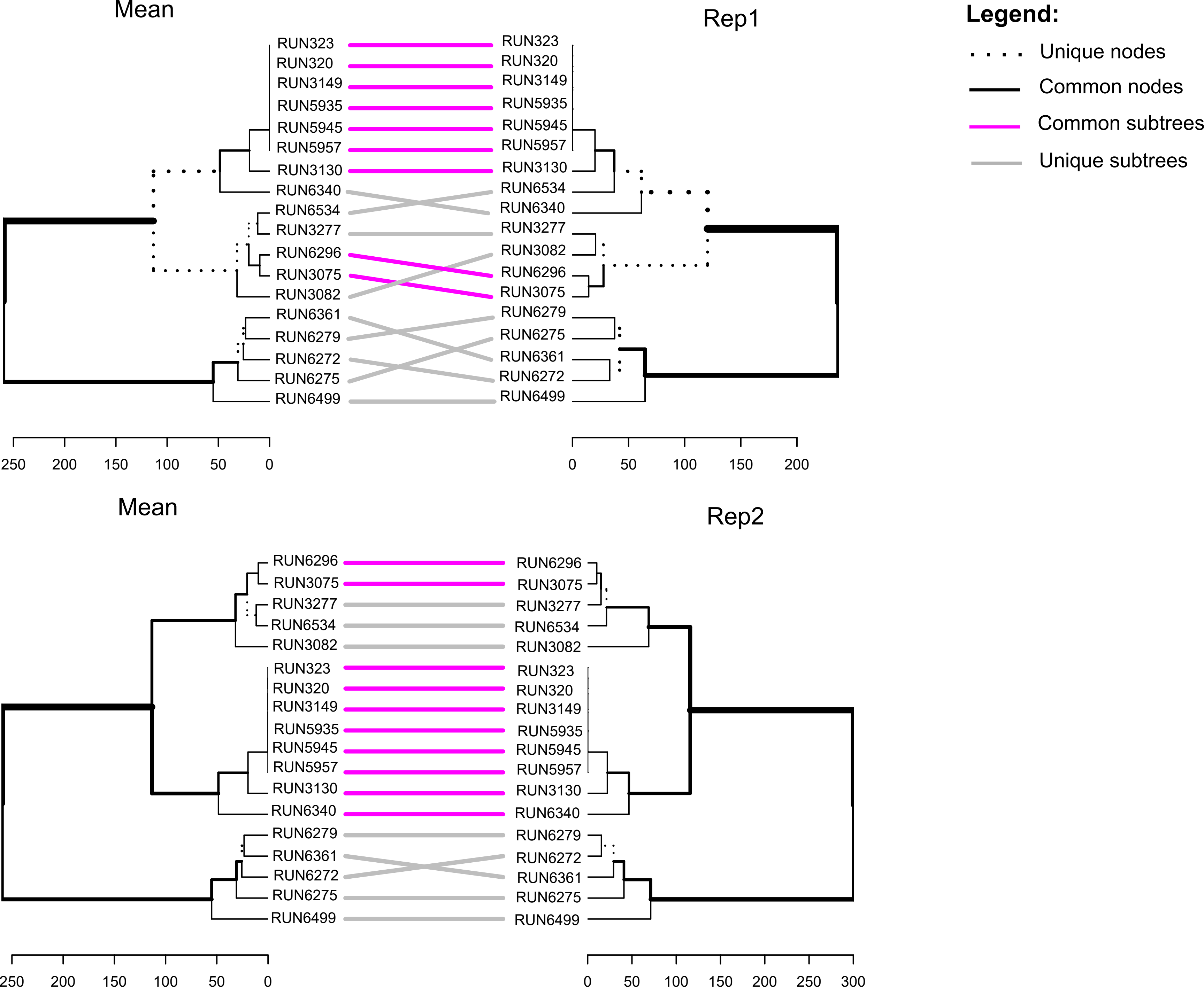
Mean
Rep1
Legend:
Unique nodes
Common nodes
Common subtrees
Unique subtrees
RUN323
RUN320
RUN3149
RUN5935
RUN5945
RUN5957
RUN3130
RUN6340
RUN6534
RUN3277
RUN6296
RUN3075
RUN3082
RUN6361
RUN6279
RUN6272
RUN6275
RUN6499
RUN323
RUN320
RUN3149
RUN5935
RUN5945
RUN5957
RUN3130
RUN6534
RUN6340
RUN3277
RUN3082
RUN6296
RUN3075
RUN6279
RUN6275
RUN6361
RUN6272
RUN6499
250
200
150
100
50
0
0
50
100
150
200
Mean
Rep2
RUN6296
RUN3075
RUN3277
RUN6534
RUN3082
RUN323
RUN320
RUN3149
RUN5935
RUN5945
RUN5957
RUN3130
RUN6340
RUN6279
RUN6361
RUN6272
RUN6275
RUN6499
RUN6296
RUN3075
RUN3277
RUN6534
RUN3082
RUN323
RUN320
RUN3149
RUN5935
RUN5945
RUN5957
RUN3130
RUN6340
RUN6279
RUN6272
RUN6361
RUN6275
RUN6499
250
200
150
100
50
0
0
50
100
150
200
250
300

Fig. S1. Collection area of RSSC phylotype I in Madagascar. Each black dot indicates the site of collection, the white circle contains information about the plot number (upper part of the circle) and the number of isolates collected in each site (lower part of the circle).

Fig. S2. Minimum spanning tree (MST) of the Malagasy phylotype I according to the samples' host. The haplotypes were identified by using goeBURST full MST in PHYLOVIZ. Each MLVA type (MT) is displayed as a circle, the size of which is proportional to the number of isolates represented. The different colours indicate the host of isolation. The branch thickness depends on the number of locus differences between the neighbouring haplotypes. MCC1 to MCC11 represent the Malagasy clonal complexes 1 to 11. A clonal complex is composed of haplotypes that differ only by one VNTR locus. Here, n represents the number of strains and G represents the number of haplotypes.

Fig. S3. Tanglegrams representing the correspondence between different dendrograms built from the inhibition activity of 18 bacteriocin-producing Malagasy isolates. The left dendrogram was built from the mean data and the right dendrogram from each of the biological replicates (Rep1 and Rep2). The dotted line represents unique nodes, the solid black - pink and gray lines represent respectively common nodes, common subtrees and unique subtrees.
